# Feasibility Study of Advanced Neural Networks Applied to sEMG-Based Force Estimation

**DOI:** 10.3390/s18103226

**Published:** 2018-09-25

**Authors:** Lingfeng Xu, Xiang Chen, Shuai Cao, Xu Zhang, Xun Chen

**Affiliations:** Department of Electronic Science and Technology, University of Science and Technology of China, Hefei 230027, China; xlfustc@mail.ustc.edu.cn (L.X.); caoshuai@ustc.edu.cn (S.C.); xuzhang90@ustc.edu.cn (X.Z.); xunchen@ece.ubc.ca (X.C.)

**Keywords:** force estimation, surface electromyography, neural network

## Abstract

To find out the feasibility of different neural networks in sEMG-based force estimation, in this paper, three types of networks, namely convolutional neural network (CNN), long short-term memory (LSTM) network and their combination (C-LSTM) were applied to predict muscle force generated in static isometric elbow flexion across three different circumstances (multi-subject, subject-dependent and subject-independent). Eight healthy men were recruited for the experiments, and the results demonstrated that all the three models were applicable for force estimation, and LSTM and C-LSTM achieved better performances. Even under subject-independent situation, they maintained mean RMSE% of as low as 9.07 ± 1.29 and 8.67 ± 1.14. CNN turned out to be a worse choice, yielding a mean RMSE% of 12.13 ± 1.98. To our knowledge, this work was the first to employ CNN, LSTM and C-LSTM in sEMG-based force estimation, and the results not only prove the strength of the proposed networks, but also pointed out a potential way of achieving high accuracy in real-time, subject-independent force estimation.

## 1. Introduction

Surface electromyography (sEMG), which originates from the electrophysiological and mechanical activation of muscle fibers in vivo, has been regarded as a reliable way of examining the activation state of muscles. Due to its ease of collection and noninvasiveness, sEMG offers versatile applications in a wide range of research fields, including myoelectric control [1], biofeedback [2], fatigue evaluation [3], and gesture recognition [4]. Furthermore, the adoption of sEMG in muscle force estimation has been a tremendous success, and to this day, an increasing number of researchers have been attracted to delve in this field, improving the performance of sEMG-based force estimation from various aspects.

In terms of computational algorithms, numerous models have been built up to map the sEMG-force relationship, including the Hill model [5], polynomial fitting model [6], fast orthogonal search (FOS) [7], and parallel cascade identification (PCI) [8]. Recently, along with the booming development of artificial intelligence, more attention has been paid to the neural network method. Choi et al. set up a one-layer fully-connected neural network with 12 hidden units [9]. The model was trained to estimate palmar pinch force and showed promising performances in subject-dependent circumstance, but failed in the subject-independent test. Bai et al. extracted the mean frequency (MF) from sEMG using a continuous wavelet transform (CWT), and modeled the MF-force relationship using a two-layer feedforward neural network [10]. In the experimental task of predicting muscle force in elbow and knee contraction, the network succeeded in catching the dynamics of four different muscles under subject-dependent circumstance. Peng et al. proposed a time delay neural network (TDNN) based method to map the isometric contraction of human shoulder [11]. Compared to the most used feedforward network, TDNN had less risk of overfitting, and performed fairly well in subject-dependent circumstance.

From the existing works, it can be seen that most researchers tended to use multilayer feedforward neural network to carry out force estimation. The networks usually performed well under subject-dependent circumstances, while in the subject-independent situation, they may malfunction due to their poor generalization ability. As for the two mainstream architectures used in today’s artificial intelligence field, namely convolutional neural network (CNN) [12] and recurrent neural network (RNN) [13], to our knowledge, there are still few researchers using them to undertake muscle force estimation.

CNN has been proved efficient in a variety of areas, including computer vision, speech recognition, and natural language processing [14]. In the sEMG-related field, it has been applied to myoelectric pattern recognition [15,16,17]. Apart from a number of applications regarding classification and recognition, CNN is also highly effective in generative tasks, such as image in-painting [18], image super-resolution [19], and image colorization [20], to name a few. Making use of convolutional filters and pooling operations, a standard CNN can extract spatial features from different positions of the input, and thereby learn the complex relationship between two sets of pictures. As the derivation of sEMG-force relationship is also based on extracting the hidden correlation between two sets of signal, there is a potential for transforming force estimation tasks into an image-to-image translation task, and achieving better sEMG-force relationship by exploiting the superiority of CNN in handling 2D images. In regard of RNN, its strength of capturing long-term dependencies in sequential data has contributed a lot to various research points such as name entity recognition [21], machine translation [22], and sentiment classification [23], making it an even more suitable method for sEMG-based force estimation, which is a typical sequence-to-sequence task.

Besides, there are studies reporting that feeding higher-level representation of input data into RNN would make it easier to learn the temporal structure between successive steps as well as to exploit more non-temporal information from the original input [24]. This finding has triggered the attempt of combining CNN with RNN, and successful examples can be found in various applications like speech recognition [25], image caption [26], and text classification [27]. Inspired by these research results, it is worth a shot to extract high-level features from sEMG signal using CNN, and then feed the feature sequence to a RNN so that better generalization ability can be achieved.

In this paper, the main goal was to explore the feasibility of undertaking muscle force estimation using CNN and RNN. Static isometric biceps brachii contraction experiments were carried out, and the finely preprocessed data were segmented in accordance with different circumstances (subject-dependent, multi-subject, and subject-independent) so as to train a standard CNN and a long short-term memory (LSTM) RNN [28]. Further, the aforementioned two models were combined into a single one, namely the C-LSTM, and its performance under different circumstances was also examined. As far as we know, this is the first time CNN, RNN and their combination has been used in sEMG-based muscle force estimation.

## 2. Materials and Methods

**Figure 1:**
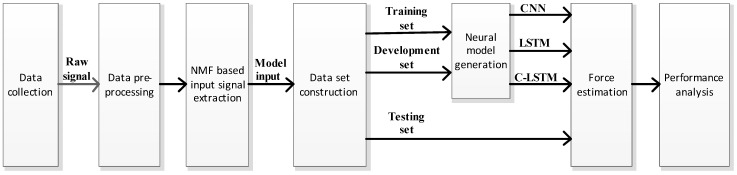
Block diagram of the proposed framework.

A block diagram of the whole force estimation framework is shown in Figure 1. In short, the scheme started from sEMG and muscle force data collection based on a high-density (HD) electrode grid and a dynamometer. Then, multi-step preprocessing was conducted, and proper data sets were constructed according to the various circumstances. Among those data sets, the training set provided information for building the networks, namely CNN, LSTM and C-LSTM, while a development set was adopted to fine tune the structures of the networks. Once the fine tuning was done, a testing set was employed to examine the final performances of different networks, and the results were compared and analyzed in order to find out their applicability to the task.

### 2.1. Subjects

Eight men (mean ± SD age 23.8 ± 2.2 years old; body mass 69.5 ± 5.8 kg; height 177.4 ± 5.3 cm) without any muscular disorder were recruited for this study. To avoid potential confounders, the age, mass and height of subjects were chosen within rather small ranges. Before the data acquisition experiment, the subjects were asked to refrain from any heavy upper limb exercises for 48 h, and abstain from any intake of caffeine for 24 h. The whole experiment was approved by Ethics Review Committee of Anhui Medical University (No. PJ 2014-08-04), and every subject signed an informed consent before the experiments.

### 2.2. Experimental Protocol

**Figure 2:**
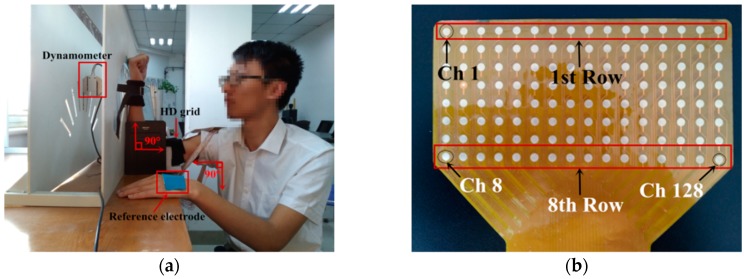
(**a**) The apparatus to aid the recording process. (**b**) The arrangement of electrodes on the HD grid.

In order to examine the feasibility of different networks on sEMG-based force estimation, an elbow flexion experiment was designed. sEMG were collected from the biceps brachii, and the elbow flexion forces were indirectly obtained from the wrist. To ensure the movement was completely undertaken by biceps brachii, the apparatus shown in Figure 2a was employed. Subjects were asked to sit upright before the desk, with the dextral upper arm positioned on the pad. Their upper arms were perpendicular to the torso, and their forearms leaned on the flat plane of the apparatus, forming another right angle. The dynamometer was fixed at the center groove of the apparatus and firmly bound to the subject’s wrist using an inelastic strap. By doing so, the wrist joint was immobilized, decreasing the influence of wrist force. Therefore, horizontal force, which was produced through the elbow flexion and measured at the wrist, could be collected as an indirect reflex of biceps brachii forces. In preparation, subjects’ skin areas beneath the HD grid were cleaned carefully with ethyl alcohol and clean water. A home-made two-dimensional HD grid was attached to the dextral biceps brachii and fastened with elastic straps so that electrodes would not deviate from muscle belly. Also, a reference electrode was pasted to the right elbow so that differential sEMG signals could be collected. Another reference electrode in connection with the ground was placed on subject's left hand to reduce 50 Hz power line interference.

**Figure 3:**
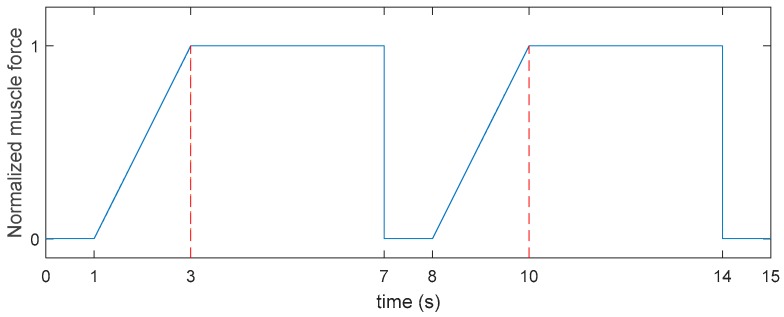
The force generation pattern in the experiment.

To complete the experiment, subjects were requested to accomplish three trials of elbow flexion at 72-h intervals, and different force levels were applied each time. At the beginning of each trial, subjects were required to finish three rounds of maximum voluntary contraction (MVC), with two minutes of rest in between. The maximum value among them would be adopted as a valid MVC. After that, subjects would perform repetitive elbow flexions according to the force generation pattern shown on the computer screen. Figure 3 presents the force pattern that subjects should follow. To stay in line with the pattern, subjects first increased their flexion force to a target level (35% MVC, 50% MVC, or 65% MVC) at constant speed within two seconds. Then, they maintained the force level for another four seconds. Following each contraction was a one-second interval, during which subjects relaxed their muscle, decreasing the force output to the baseline. The repetitive elbow flexion continued until subjects were incapable of achieving and stably maintaining the target force level for three cycles in a row.

As for the detail of data acquisition facilities, the arrangement of electrodes on the HD grid is shown in Figure 2b. The grid covered a skin area of 13.2 cm × 6.3 cm and included 128 electrodes. Each electrode had a diameter of 3.5 mm, with inter-electrode distance of 8 mm. The sampling rate of sEMG and force was 1000 Hz based on 16-bit A/D converter (ADS1198). Raw sEMG data were amplified 1371.1 fold and filtered with the bandwidth of 20–500 Hz. Then, pre-processing was carried out in Matlab R2016b. Later, force estimation models were implemented by Python 3.6, with support of TensorFlow, and the training process was conducted using NVIDIA GTX 1080 Ti.

### 2.3. Signal Pre-Processing

**Figure 4:**
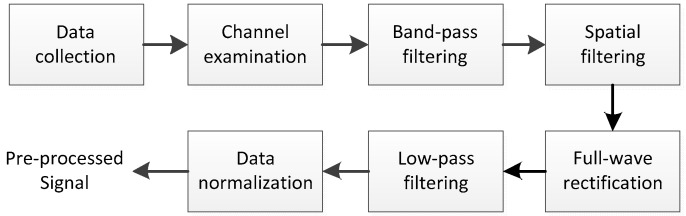
Block diagram of the pre-processing stage.

In this work, several steps of signal preprocessing were undertaken in order to remove noises and unnecessary components. The whole process can be seen in Figure 4. In the first place, the collected 128-channel sEMG data were examined carefully, as there were usually two to four channels that produced abnormal outputs. Those channels would be replaced by randomly picked neighboring channels. Next, a 20–500 Hz band-pass filter (window-based finite impulse response filter, Hanning window, 50th order) was applied to remove low frequency noise and motion artifacts. Then, we employed principle component analysis (PCA) to act as a spatial filtering method. As suggested in previous research [29], the first and the last few modes of the PCA-processed signal might contained either redundant common information or measurement noise, and discarding them would be beneficial for producing accurate force estimation. Referring to our former study that shared the same apparatus and similar data pre-processing [30], the first two modes as well as the last two modes were removed at this stage. After PCA processing, the remaining modes were reconfigured to form 128-channel elaborately polished sEMG signal. Finally, full-wave rectification and 5 Hz low-pass filter (window-based finite impulse response filter, Hanning window, 50th order) were adopted to extract the envelope of each channel. Both the measured force and the extracted envelopes were normalized by their maximum values in each contraction cycle.

### 2.4. Force Model Input Signal Extraction Based on NMF Algorithm

So far, normalized 128-channel sEMG as well as force data were obtained. In addition, as the sEMG were originally collected by HD grid, it was possible to further optimize the signal by selecting the most appropriate region for force estimation. To this end, a nonnegative matrix factorization (NMF) based optimization scheme was employed, which had been proved successful in reducing electrodes redundancy and screen out channels contributing positively to the force pattern [30,31,32].

Taking use of the scheme, sEMG were initially decomposed into the linear combination of several activation patterns recruited by corresponding time-varying coefficient vectors. As shown in formula (1), non-negative matrix $M\in R^{m\text{×}n}$ represented the multi-channel normalized sEMG signal (*m* channels and *n* sample points), while non-negative matrices $W\in R^{m\text{×}s}$ and $C\in R^{s\text{×}n}$ (noted that *s* < *m*) stood for activation patterns and time-varying coefficients. Each column of *W* represented an activation pattern with *m* weighting factors. Those factors described the relative contribution of every channel to the corresponding row of *C* that specified how the activation pattern was modulated across the movement cycle:

(1) $$M^{m\times n}=W^{m\times s}C^{s\times n}.\;$$

Then, activation intensities were computed according to Equation (2):

(2) $$Intensity\left(i\right)=\sum_{t=1}^{n}C_{i}\left(t\right),\;$$

where *i* ranged from 1 to *s*, and *n* is the length of the sample. In this work, *s* was set to 2, for our previous study has suggested that two activation patterns were sufficient to explain most of the sEMG variance [30].

After the calculation of Equation (2), the activation pattern corresponding to the highest *Intensity* was taken as the major activation pattern. According to our former study, the channels whose weighting factors ranked the first quarter in the major activation pattern contributed the most to the force pattern, and should be considered most suitable for force estimation [30]. Thus, the first 32 channels that had larger weighting factors were sorted out from the major activation pattern in this work, and the average of those normalized sEMG was saved for later use.

### 2.5. Data Set Construction

Neural methods are data-hungry, and would have poor generalization ability if trained in low-resource situations. Previous research on sEMG-based force estimation had also suggested this idea, showing that neural networks trained on small data sets tended to fall flat in subject-independent circumstances [9].

For this work, the problem became much more serious, since our data come from multiple experiments and different force levels. As depicted in Figure 3, our data consisted of many contraction cycles. If each cycle was simply considered as a sample, we would end up with a fixed- pattern, less-informative, and extremely small data set. A network trained on such a data set could hardly have any generalization ability, and it would be impossible for it to be applied on real-time estimation. Therefore, a proper data set construction strategy must be undertaken, in order to increase the training efficiency and improve the networks’ generalization performance.

**Figure 5:**
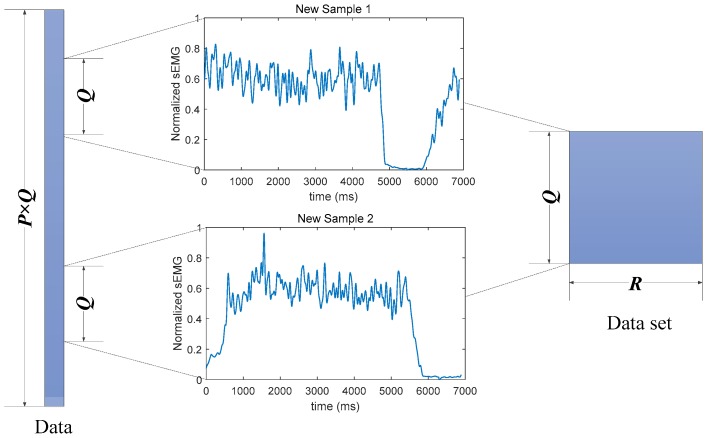
Data set construction process.

Here, the data were divided into a training set, development set, and testing set. A random truncation strategy was adopted to build up each set for the sake of including more information and real-time application. Taking the training set for example, supposing *P* contraction cycles were allocated to this data set, and each contraction cycle had *Q* points, then its length should be *P* × *Q*. Next, *R* uniformly distributed random integers were generated within the range of [1, (*P* − 1) × *Q*]. Correspondingly, a new training set with *R* samples could be built by extracting the *Q*-point data segments starting from each of the generated numbers. The whole process was diagrammatized in Figure 5, and applied to development set and testing set.

### 2.6. Neural Networks for Force Estimation

Three neural networks were exploited in this paper, namely a standard CNN, a standard LSTM, and a C-LSTM, which combines CNN and LSTM together. In this part, the basic theories of CNN and LSTM were provided, along with the basic structures of each model.

As mentioned in the Introduction section, CNN has a wide range of applications. Among them, computer vision turns out to be the area that CNN is especially adapted to. 2D convolutional filters deployed on multiple layers make it possible to hierarchically extract local representations. Pooling operation allows the network to gradually see larger portion of the input, while also provides a degree of translation invariance, making it less affected by small variations in position [33].

In general, let ⊗ be the symbol of convolution calculation, the output produced by the *l*-th layer of CNN can be written as Equation (3), where *X^l^* and *X^l^*^−1^ are the feature maps at layer *l* and *l* − 1 respectively, while *w* and *b* are referred to as weights and bias. *f* is activation function, usually being set as Rectified Linear Unit (ReLU) [34]:

(3) $$X^{l}=f\left(w^{l}⊗X^{l-1}+b^{l}\right),\;$$

Following each convolution, pooling is usually carried out to increase the network’s receptive field size, or to add a degree of translation invariance. One of the most commonly used pooling operations is max-pooling [35]. In short, this operation divides the input image into a set of non-overlapping rectangles and outputs the maximum value of each rectangular region.

**Figure 6:**
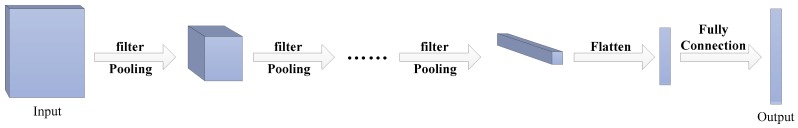
Structure of the standard CNN [12].

Figure 6 presents the architecture of a typical multi-layer CNN. Exploiting the idea of transforming force estimation into an image-to-image translation task, when applying CNN to our task, the input *Q*-point sample was reshaped to a square image. Then, a series of convolution followed by max-pooling were carried out. The outcome was flattened and encoded using a fully connected layer to form a *Q*-point force estimation output.

In contrast with CNN, the recurrent neural network (RNN) is generally more suitable for sequential task, in which long-term dependencies are learned by the network’s chain-like architecture [13]. However, standard RNN is not easy to train, as it is vulnerable to the problems of gradient vanishing and explosion due to the repeated multiplication of the recurrent weight matrix [36]. To address these problems, several variants of RNN have been put up, and LSTM is generally regarded as a successful one [28].

**Figure 7:**
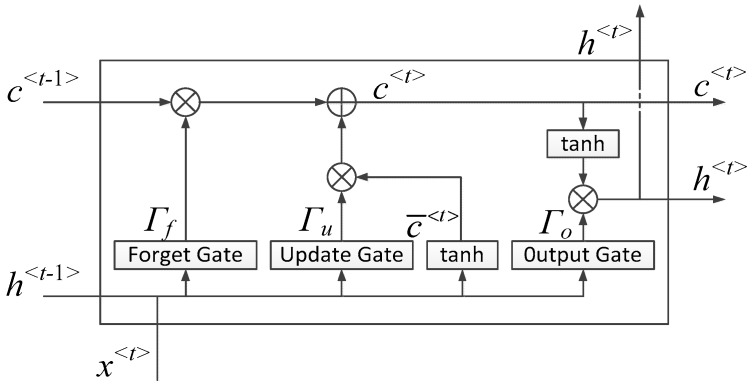
A standard LSTM unit [33].

A typical LSTM unit is illustrated in Figure 7. In the figure, *x*^<*t*>^ is the input at time step *t*. The output hidden state *h*^<*t*>^ and memory cell *c*^<*t*>^ are decided by previous hidden state *h*^<*t*−1>^, previous memory cell *c*^<*t*−1>^, and the input *x*^<*t*>^. Three gates, namely forget gate, update gate, and output gate are also calculated in the unit. Their functions can be summarized as: *Γ_f_* decides to what extent the information of old memory cell should be discarded, *Γ_u_* determines how much information should be stored in the new memory cell, and *Γ_o_* controls the output based on *c*^<*t*>^. For the sake of concision, the detailed calculation is not described here, and it can be easily found in a number of related works such as in [33]. When LSTM was employed in this work, the input was *Q*-point sequential signal, which would be fed into the network in *Q* timestamps, while the output of LSTM was encoded to be the same length as input through a fully connected layer.

Finally, a typical C-LSTM can be attained by simply stacking convolution layers and LSTM layers. As mentioned in the Introduction section, the motivation for building a C-LSTM lay in the finding that feeding higher-level representation of input data into the sequential model could boost its performance to some degree [24]. In addition, CNN had been proved effective for extracting high dimensional features from input data [37]. Therefore, it was very natural to consider adding convolution layers in front of the LSTM so as to improve its learning ability. When utilizing C-LSTM in this work, the *Q*-point input sequence was first fed into a convolution layer, where convolution calculation with stride was undertaken. Then, the extracted higher-level feature map would go through a LSTM layer, producing prediction output. Finally, the output would be flattened and encoded to *Q*-point estimated force through a fully connected layer.

### 2.7. Loss Function and Performance Assessment

In such a regression problem, appropriate loss function has to be chosen so as to drive the estimated force closer to the measured force. To this end, percentage root mean square error (RMSE%) was taken as the loss function for the models in this work. The index can be computed according to Equation (4), and is also taken to measure the model performance:

(4) $$RMSE\%=\sqrt{\frac{\sum_{i=1}^{n}{\left[F\left(i\right)-\hat{F}\left(i\right)\right]}^{2}}{\sum_{i=1}^{n}F{\left(i\right)}^{2}}}\times 100\%,\;$$

where *n* is the sample length, *F* and $\hat{F}$ are the measured force and predicted force, respectively.

## 3. Results

In the beginning of this section, the data allocation strategies for different circumstances (multi-subject, subject-dependent, and subject-independent) were described. Then, the detailed structure of each neural network was specified, along with the fine tuning methods applied to the models. Next, we discovered the necessity of employing random truncation strategy in data set construction process, and confirmed the speculation that models trained on more informative data set would achieve higher generalization ability. Finally, all results obtained under different circumstances were presented and briefly discussed.

### 3.1. Data Allocation Strategy

As mentioned in the Introduction, to this day, there are few studies regarding force estimation in subject-independent situations. Instead, most researchers tend to focus on multi-subject and subject-dependent circumstances. In order to fully explore the applicability of the proposed networks on force estimation, in this work, we aimed to examine their performances in not only multi-subject and subject-dependent circumstances, but also subject-independent situations. The data allocation strategies were as follows: In the subject-dependent circumstance, the data of a given subject were shuffled in the first place. Then, 80% of the data were taken for training, and the remaining 20% were divided in half for developing and testing. Similar process was adopted in multi-subject situation, where 80% of each subject’s shuffled data were extracted and mixed up for training, while another 10% were picked out and mixed for developing. As for the remaining 10% from each subject, they would be kept separate and taken as testing data. When it comes to the subject-independent situation, a leave-one-out technique was employed. Each time, a certain subject’s data were selected for testing, whereas the remaining seven subjects’ data were divided by the ratio of 90% and 10% for training and developing. After data allocation was carried out, a random truncation strategy was applied, generating training, developing, and testing sets of sizes 50,000, 100, and 100 from the corresponding data.

### 3.2. Detailed Structures of the Proposed Networks

For each type of network, 17 possible structures were generated after training and fine tuning, eight from the subject-dependent circumstance, eight from the subject-independent circumstance, and one from the multi-subject circumstance. To be specific, in the process of generating a candidate structure, the input sample length *Q* was set to 6889 (the square of 83) in order to stay consistent with the length of a contraction cycle (approximately 7 s) while also enable the sample to be reshaped into a square image. Batch normalization [38] and dropout [39] techniques were employed. The former helped to speed up network convergence by dynamically normalizing the inputs, while the latter introduced noise into the network to prevent overfitting. Also, early-stopping method was used. Based on the method, the training would stop once the estimated error of development set stayed increasing for six epochs in a row, and the model parameters at the epoch of minimum developing error would be taken as valid. In addition, hyperparameter (numbers of layer, filter, and hidden state; filter size and dropout rate, etc.) tuning was conducted to make sure the structure could perform well at least under a particular circumstance.

**Figure 8:**
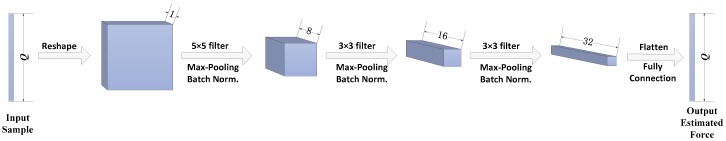
Structure of the proposed CNN.

**Figure 9:**
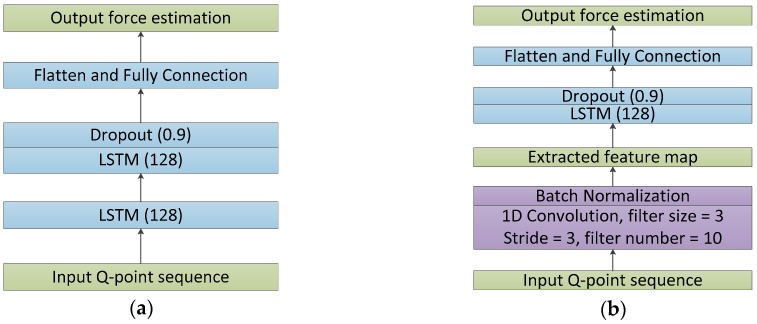
(**a**) The proposed LSTM. (**b**) The proposed C-LSTM.

We determined the final settings for each network by applying its candidate structures to all circumstances and keeping the structure that was both simple in shape and high in average performance. Figure 8 and Figure 9 depict the final structures of CNN, LSTM, and C-LSTM models in this work.

### 3.3. Estimation Results Under Different Training Strategies

Before reporting the whole results, it is significant to provide more details about how we figured out the final training strategy: employing random truncation strategy and involving multiple force levels in the training data. Here we took subject-dependent situation as example and conducted two auxiliary studies on subject 1.

#### 3.3.1. Fixed-Pattern Training vs. Varied-Pattern Training

**Figure 10:**
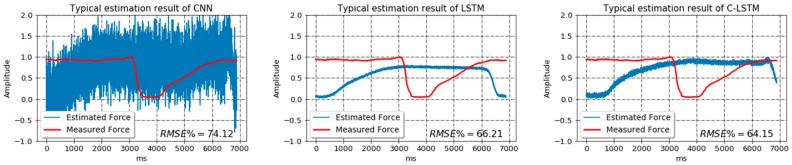
The typical force estimation output of models trained on fixed-pattern data set.

**Figure 11:**
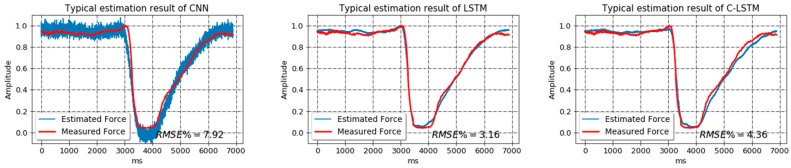
The typical force estimation output of models trained on varied-pattern data set.

In the first study, the training data were directly taken as training set, without random truncation method being applied, and so did the developing data. As indicated in the Methods section, this strategy would bring about a pattern-fixed, less-informative, and extremely small data set. In fact, we only got less than 200 samples in the training set and several dozens of samples in the development set. Once the fixed-pattern training was completed, 100 testing samples were normally generated using random truncation method from the testing data and adopted to validate the models. A fixed-pattern estimation example was shown in Figure 10 based on a typical testing sample. Additionally, the networks were also trained on a data set that was processed by the random truncation method, which we called varied-pattern training, and validated on the same testing set. In this case, the sizes of each set were 50,000, 100, and 100. Figure 11 presents the estimation trajectories of the same typical sample.

In total, the average testing RMSE% of models trained on fixed-pattern data set were 53.32%, 45.67% and 43.15% corresponding to CNN, LSTM and C-LSTM. On the contrary, the average RMSE% of models trained on varied-pattern data set were 8.25%, 5.89% and 5.64% for each model. It could be observed that when trained on the fixed-pattern data set, all models demonstrated unacceptably high testing RMSE%. However, after the training data were processed by the random truncation strategy, although the testing samples had totally different shapes compared to the normal contraction cycles shown in Figure 3, the trained networks could still identify the force patterns and perform low-error predictions. These results suggest that the training samples pattern will have a strong influence on the generalization ability of the model. A network trained on data set that only contains standard contraction cycles will learn nothing but to identify a certain type of force pattern, making it impossible to be put into real-time, multi-pattern application. Therefore, it is altogether fitting and necessary to employ random truncation strategy in data set construction stage.

**Figure 12:**
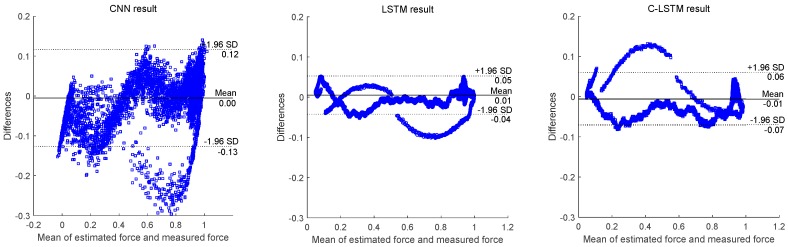
The Bland-Altman plots of the differences between the measured and estimated forces produced by different models.

As for the relative performance of different networks, it appears that the estimation trajectory of CNN tends to oscillate severely, which significantly downgrades its performance. Based on Figure 11, the Bland-Altman plots [40] of the differences between the estimated and measured forces across different models are drawn and displayed in Figure 12. It can be revealed from the figure that generally all models show a degree of consistency between estimated and measured forces by incorporating the majority of the difference values into the 95% confidence interval. Also, the mean value of difference for each model stays around zero, suggesting that all models can produce force prediction in rather high accuracy. Considering the width of 95% confidence interval, C-LSTM and LSTM present compatible performances, as most of their differences values fall in a relatively narrow range. However, when it comes to CNN, its difference values spread in a much wider area, implying it to be a less stable network for the task.

#### 3.3.2. Single-Force Training vs. Multi-Force Training

After discovering the necessity of employing random truncation strategy, in the second study, the influence of training force levels on the models’ generalization ability was examined. As shown in the Method section, it was speculated that including more force levels in the training data would contribute to the model generalization ability. The data of subject 1 were first divided into three subsets corresponding to three force levels. Then, each subset was further partitioned with the ratio of 80%, 10%, and 10%. The larger part of each subset was taken for training, while the remaining parts served as development data and testing data. To keep pace with the conclusion acquired in the former part, random truncation strategy was adopted, and the sizes of generated training, developing and testing sets were still 50,000, 100, and 100.

After data set construction, three subsets representing different force levels would take turns for training and testing the networks. Besides, the model trained in the former part based on random truncation strategy was also brought to this study and validated using different subsets, since it was trained on a data set that included all force levels. The testing results of each subset on each model were compared in Table 1. For the model trained using multiple force levels data, no matter which subset was chosen for testing, it remained stable and produced highly accurate force estimation. However, the models trained on single force level data yielded relatively worse performances. In general, they could only maintain effective when the data of training and testing came from the same force level. Once they were applied to cross-force-level testing, most of them fell behind the multi-force trained models by nearly 2% in RMSE%, clearly showing the loss of generalization ability.

Comparing the performances of different networks, it can be identified that CNN demonstrated the lowest prediction accuracy in all testing trials. This phenomenon is consistent with what has been found in the former part. C-LSTM seems to achieve the highest accuracy in most testing trials. However, since the RMSE% differences between LSTM and C-LSTM are trivial, we still consider their performances to be compatible.

In summary, the results in Table 1 confirm our assumption that more force information would contribute to better generalization ability, implying the necessity of employing data collected from multiple force levels in the model training process. Combining the conclusions drawn from the two auxiliary experiments, we had every confidence to believe that a varied-pattern, multi-force training strategy could lead to much better results.

### 3.4. Force Estimation Results Across Eight Subjects

**Figure 13:**
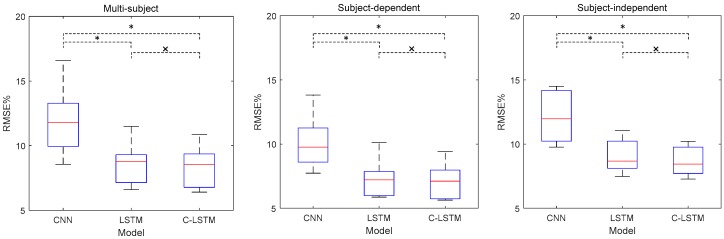
Boxplot graphs of RMSE% for each proposed model under each circumstance.

Here, we provided the overall results of CNN, LSTM and C-LSTM under three circumstances. Figure 13 depicts the results in RMSE%. To be more specific, the mean RMSE% for each model under multi-subject circumstance were 11.89 ± 2.57, 8.57 ± 1.61, and 8.32 ± 1.59. Under subject-dependent circumstance the RMSE% were 10.10 ± 1.99, 7.27 ± 1.44, and 7.09 ± 1.38. For subject-independent circumstance, the RMSE% were 12.13 ± 1.98, 9.07 ± 1.29, and 8.67 ± 1.14.

Also, the Kruskal-Wallis one-way analysis of variance was engaged. The outcomes are presented in Table 2 and annotated in each boxplot graph. As shown in the figures, ∗ represents that significant differences with *p* < 0.05 are found, while × indicates that the differences are insignificant. From Figure 13, a conclusion similar to what was found in the auxiliary study can be drawn, suggesting that CNN was the worst method for the task. As for LSTM and C-LSTM, the results also regarded C-LSTM as slightly more robust than LSTM, but the improvements were far from significant.

## 4. Discussion

In this paper, we mainly examined the feasibility of using three types of neural networks in force estimation across three circumstances. Static isometric contraction of the biceps brachii was taken as an exemplary task, and the results suggested that all models were applicable, with two of them being especially effective. Before going on to discuss the performances and limitations of the proposed networks, there are two points that we feel like emphasizing.

The first point is the inclusion of subject-independent situation, which was rarely noticed in previous studies. As shown in the Results section, the models in this work achieved high accuracy under subject-independent circumstances. This outcome not only proved the efficiency of advanced neural methods, but also drew them closer to utility. Since force estimation plays a crucial role in myoelectric control systems, a model that can adapt to numerous users without being retrained can certainly enlarge the systems application range.

Another point that deserves attention is the significance of varied-pattern, multi-force training. It was demonstrated in the former section that fixed-pattern training would bring about a model that was only capable of identifying a certain force pattern, greatly limiting its generalization ability. Similar conclusion could also be drawn from the results of single-force training. As sEMG-force relationship can be different across various force levels, a model trained based on only a given force level data can hardly be versatile. Hence, here we underscore the necessity of including as many patterns and force levels as possible in the training process, which may contribute to both higher prediction accuracy and real-time application.

Now we remark the performances of the models. Generally, compared to LSTM and C-LSTM, CNN yielded the worst performance in every circumstance, providing much more blurry outputs than other networks. This outcome suggests that simply transforming a force estimation into an image-to-image translation task without modifying the standard CNN structure is not enough, as it may only learn the large-scale relationship and fail to capture the subtle correlation. To achieve better result, more in-depth improvement should be carried out on the traditional CNN, including putting up new loss function other than RMSE% to guide the model’s convergence and improving the basic structure of the network in order to match the force estimation task. One example can be changing the activation function from ReLU to Leaky ReLU [41], which is widely used in generative models. Also, taking references from the numerous researches in computer vision, it is reasonable to employ some useful structures, like the U-Net [42], for instance, to upgrade the CNN’s ability of generating stable output. Finally, we can turn to some other much complicated structures for help, and a mostly possible choice can be Generative Adversarial Networks (GAN) [43] as well as its variants [44]. GAN has been proved successful in solving generative problems for many years [45], and it may be more adaptive to the task of force estimation.

As for LSTM and C-LSTM, their relatively better performances have justified the use of sequential structures in force estimation tasks. Judging from the boxplot graphs in Figure 13, the overall performance of C-LSTM was slightly better than that of LSTM, implying that to some extent a higher-level representation of input data can indeed improve the prediction accuracy. However, as this phenomenon is not obvious, it can be inferred that only adding an unmodified convolutional layer to extract high dimensional representation may not be enough to make significant difference. Therefore, more structure improvement should be conducted on the front part of C-LSTM, and the methods mentioned in the former paragraph can also be adopted here.

Apart from the deficiency in structure, muscle fatigue can also be counted as a factor that affected the performances of neural networks. As mentioned in the Methods section, the data acquisition experiment would continue until the subject could no longer provide a usable output. Thus, it was inevitable for muscle fatigue to be involved. Although the networks trained on those data seem to work all right, the influence of muscle fatigue remained unclear. It will be our follow-up study to undertake comparative experiments and analyze the actual effects of muscle fatigue on the neural networks.

In the last part, we address the limitations that may hinder the models in practical use. Perhaps the most obvious limitation lies in the versatility of the networks. In this work, we only took a certain type of movement to examine the proposed networks. However, human motion is a complex process, which calls for the combination of multiple muscles and voluntary movement patterns. Thus, in a practical situation, the force estimation task is likely to be more complex than in this exploratory study. If the networks cannot adapt to further complicated movements, their application would be seriously limited. Also, to settle the differences between muscle characteristics, far more subjects across different sizes, sexes, and ages will be expected to complete an initial data base, and the hardship of data collection can be another problem. Finally, even if enough data are acquired, the cost for training and optimizing the models can never be neglected, since neural networks are usually hard to train and tune.

Considering these limitations, it will be necessary to come up with proper network structures that can generalize across different tasks, and to figure out a method that can maximize the information of data available. For the first goal, we will set out to evaluate the networks in more complicated scenarios, such as varied speed isotonic contraction or random contraction, and try to modify their structures taking use of the aforementioned knowledge. For the second goal, more subjects apart from young males must be recruited in order to increase the information of our data base, and we may seek inspiration from the field called machine teaching [46], whose aim is to design a minimal training set and find an optimal training sequence to steer the model's learning process.

## 5. Conclusions

In short, this paper examined the feasibility of CNN, LSTM and C-LSTM in the application of sEMG-based muscle force estimation. Promising results were found in LSTM and C-LSTM across three different circumstances, while CNN appeared to be less stable. The investigation also suggested that proper data processing techniques and the involvement of multiple force levels could contribute a lot to the generalization ability of the proposed models. As far as we know, this work was the first to introduce CNN, LSTM and C-LSTM to map sEMG-force relationships, pointing out a way to achieve better force estimation in real-time, subject-independent situations.

## Figures and Tables

**Table 1:** Testing RMSE% of subsets on models trained using single-force and multi-force data.

	Testing	Models	35% MVC	50% MVC	65% MVC
Training					
35% MVC		CNN	8.38	10.53	8.53
		LSTM	5.80	7.57	7.02
		C-LSTM	5.84	7.56	6.86
50% MVC		CNN	9.73	8.44	7.33
		LSTM	8.89	6.38	6.16
		C-LSTM	9.29	6.31	6.04
65% MVC		CNN	10.93	9.36	7.55
		LSTM	9.33	8.50	6.16
		C-LSTM	9.74	7.65	5.95
Multiple Force levels		CNN	8.22	8.53	7.23
		LSTM	6.24	5.87	5.81
		C-LSTM	6.10	5.69	5.22

**Table 2:** Kruskal-Wallis analysis on the overall results of CNN, LSTM and C-LSTM.

	Circumstances	Multi-Subject	Subject-Dependent	Subject-Independent
Models				
CNN vs. LSTM		0.0157	0.0063	0.0087
CNN vs. C-LSTM		0.0087	0.0063	0.0023
LSTM vs. C-LSTM		0.6744	0.6744	0.4008
